# Scientific and regulatory progress in advancing paediatric oncology drug development in the EU and in the US

**DOI:** 10.3389/fmed.2025.1642279

**Published:** 2025-09-26

**Authors:** Giorgio Reggiardo, Alessandra Natale, Nicola Santoro, Viviana Giannuzzi, Claudia Pansieri, Mariagrazia Felisi, Donato Bonifazi, Adriana Ceci

**Affiliations:** ^1^CVBF-Consorzio per le Valutazioni Biologiche e Farmacologiche, Bari, Italy; ^2^TEDDY-European Network of Excellence for Paediatric Research, Pavia, Italy; ^3^Paediatric Onco-haematology Unit, Aldo Moro University of Bari, Bari, Italy; ^4^FGB-Fondazione per la Ricerca Farmacologica Gianni Benzi Onlus, Bari, Italy

**Keywords:** paediatric oncology, paediatric tumours, mechanism of action (MOA), molecularly targeted drugs, immunotherapies, paediatric regulation

## Abstract

**Introduction:**

This study provides an updated overview of progress in paediatric oncology, following legislative and regulatory initiatives in the European Union (EU) and in the United States (US). In particular, the US Research to Accelerate Cures and Equity (RACE) Act 2017 mandated new paediatric indications based on drug Mechanism of Action (MoA), and the EE 2015 revision of the waivers system allowed more agreed Paediatric Investigation Plans (PIPs).

**Materials and methods:**

The sample included: (a) products with paediatric oncology marketing authorisation in the US and in the EU from 2007 to 2024; (b) PIPs granted in the EU during the same period. Linear regression models were used to evaluate the time-trends in approvals, and the chi-squared test was applied to compare categorical variables in the periods ranging between 2007–2017 and 2018–2024.

**Results:**

In the 2018–2024 period, more paediatric products have been approved in both regions, with the US progressing at a rapid pace. The approved indications for solid tumours (STs) are growing, with innovations from targeted and immunotherapeutic agents prevailing over chemotherapies. The approval of PIPs reflects a similar trend to that of the products. Both paediatric products and PIPs are granted mainly to address specific childhood tumours, rather than those derived solely from adult indications. However, several unmet needs remain to be addressed.

**Discussion:**

Both regions are working to advance paediatric oncology medicines. However, a significant gap still exists between the EU and the US, with the EU lagging behind. This discrepancy should be a priority for Europe. It is unlikely that abolishing the Paediatric Regulation, proposed as part of the Pharmaceutical Legislation reform, in the absence of other initiatives and substantial investments, would be the right solution.

## 1 Introduction

Both in the European Union (EU) and the United States (US), existing Paediatric Regulations require pharmaceutical companies that are developing a new active substance (AS) or a still in-patent medicinal product (MP), to apply and agree with the Regulatory Bodies [the US Food and Drug Administration (FDA) and the European Medicines Agency’s Paediatric Committee (EMA-PDCO)] specific paediatric drug development programmes. Such programmes, including the Paediatric Investigation Plan (PIP) in the EU and Paediatric Study Plan (PSP) in the US, require data supporting the paediatric use of the MP to be provided at the time of a marketing authorisation (MA), unless a waiver is granted by the Agencies (1–3). During the first years of the EU Paediatric Regulation’s application, the number of waivers granted due to the ASs indication for a condition occurring in adults only prevailed over the number of approved PIPs (4, 5). This led to poor development of new paediatric medicines compared to the adult ones, especially in some therapeutic areas, including paediatric oncology (4). In particular, very few PIPs were approved for paediatric solid tumours due to the notable differences between childhood and adult tumours. In the US, paediatric rules up to 2017 have been largely ineffective for oncology products, as they granted an automatic waiver from the obligation to develop a PSP for rare diseases (3, 4, 6). Moreover, most paediatric tumours are rare conditions, where drug development is primarily hindered by safety and efficacy concerns (7, 8).

More recently, the identification of relevant targets and pathways for some adult cancers has offered new approaches for effective treatments of various tumours. Since a vast number of novel ASs targeting genetic mutations and molecular pathway aberrations, such as MYCN, BIRC5, PHOX2B, epigenetic factors, tyrosine receptor kinase (TRK) inhibitors, and other targets, have been demonstrated to be relevant for paediatric oncology diseases (9, 10), several of these ASs are expected to be developed for paediatric use (10). Because several key oncogenic driver mutations are shared across multiple tumours regardless of the location or histological feature, selective agents, known as ‘tissue-agnostic’ treatments (11), are designed to target these mutations across multiple cancer types (12). These advancements may represent an advantage in reducing the burden of multiple trials conducted in a small and limited population. Advances in paediatric care are also expected from gene therapy, cellular immunotherapy (CAR-T cells), and neoantigen-based cancer vaccines, especially from the messenger RNA (mRNA) vaccines representing a new, promising frontier that has received input from the successful generation of two mRNA vaccines in response to the severe acute respiratory syndrome coronavirus 2 (SARS-CoV-2) pandemic (13). Apart from the most recent innovations, few new therapies have been approved for paediatric use and particularly for solid tumours (11, 14). For this condition, drug development is minimal due to the proven difficulties in targeting cancerous cells correctly, regardless of adult tumour targets (15–17).

Additionally, paediatric oncology products, like other products for children, encounter various research barriers and gaps that hinder their development. These include the unique biology of paediatric tumours, which necessitates tailored research and therapeutic strategies for children; the rarity of paediatric cancer diagnoses, which limits trial enrolment; and limited investments from sponsors, often due to low economic interest and market returns (16, 17). This results in a lengthy, complex, and often delayed approval process compared to adult products, with a median time between the first-in-human trial and first-in-child trial equal to 6.5 years, which may extend for as long as 28 years in exceptional cases (18). Consequently, while there has been a considerable increase in the number of adult MPs approved by the EMA since its establishment, the average annual growth of paediatric MPs has remained significantly lower (4). For instance, across the period from 2007 to 2019, of a total number of 1,190 medicinal products approved by the EMA, only 34% (405) were paediatric, with a total of 22 belonging to the oncology therapeutic area. Of those, very few were targeted or immunotherapeutic drugs (4).

To integrate scientific advancements and expedite pediatric drug development in both the EU and the US, existing paediatric legislative initiatives (1, 19) have been updated recently (20, 21).

More specifically, in the US, the 2017 ‘Research to Accelerate Cures and Equity for Children Act’ (RACE) (19) requires that oncological products developed for adult indications must also be considered for paediatric development if their mechanism of action (MoA) is relevant to paediatric cancers. For the effects of the RACE Act, it is expected that oncology products addressing paediatric therapeutic needs and targeting paediatric-specific molecular markers will prevail over drugs derived solely from those already developed for adults. The RACE Act also eliminated the automatic waiver applicable to medicines for rare oncologic diseases like paediatric tumours.

In the EU, as of July 2015, the Paediatric Committee (PDCO) adopted a review of the class waiver list (22), which finally entered into force in 2018 and aimed at restricting any types of automatic waiver applications for new MAs (23). At the same time, to bring new paediatric anticancer drugs more rapidly to the market, scientists and paediatric oncology Networks demanded that, also in the EU, the drug development for children and adolescents with cancer should meet an MoA-based criterion rather than being driven only by the adult condition (24–26).

Moreover, in 2016, the European Parliament issued a resolution (27) for addressing a substantial revision of the Paediatric Regulation, stating that ‘the regulation has not sufficiently addressed areas with the greatest paediatric need for medicines,’ as demonstrated by the fact that the majority of the approved paediatric indications have been ‘driven’ by adult condition, while very few investments have been devoted to innovative medicines covering paediatric-specific diseases (non-adults-driven) and to implement new Paediatric Use Marketing Authorisation (PUMA) (28, 29).

The lack of drugs addressing children’s specific needs has also been a primary concern within the scientific and patient paediatric oncology community, and has been part of the official debate on the revision of the Paediatric Regulation proposed by the European Commission (30). In that regard, SIOPE—The European Society for Paediatric Oncology strongly advised the ‘inclusion of a first-in-child marketing authorisation incentive to foster commercial interest in developing medicines specifically for paediatric cancers and rare diseases’ (31).

Thus, given this scenario, this study focuses on two objectives. First, it aims to assess the progress of paediatric oncology drugs in the EU by considering the number and the annual trend of MPs approved in the period 2007–2024 compared with the paediatric oncology approvals in the US in the same period. Differences between haematological malignancies and solid tumours were considered.

Second, we aim to evaluate whether paediatric oncology drugs were developed from an existing adult indication (adult-driven) or to address a paediatric-specific oncology indication (non-adult-driven).

Finally, the drugs were divided into three categories (e.g., cytotoxic-chemotherapeutic, molecularly targeted, and immunotherapy) to appreciate the contribution of innovative targeted therapeutics to address unmet paediatric cancer care.

To capture the possible effects of the ongoing scientific and regulatory changes in the US and EU, two periods were compared: 2007–2017 and 2017–2024.

## 2 Materials and methods

### 2.1 Sample

The sample included the following:

MPs received a marketing authorisation for a paediatric oncology indication in the EU and the US in the period from 2007 to 2024.

PIPs submitted and granted by the EMA-PDCO for a paediatric oncology indication from 2007 to 2024.

Sources. The list of Paediatric oncology MPs approved in the EU has been extracted from the European Paediatric Medicines Database (EPMD), a standardised database created in 1996 to collect paediatric MPs approved in the EU, including general and developmental details.^1^ The EPMD, managed by TEDDY—European Network of Excellence for Paediatric Research, is continuously updated by deriving information from EMA official sources.^2^

FDA-approved paediatric oncology MPs were derived from the Paediatric Oncology Drug Approvals file and its accompanying downloadable document, “Pediatric Approvals Additional Information” (updated November 2024 and available at https://www.fda.gov/about-fda/oncology-center-excellence/pediatric-oncology-drug-approvals).

The analysis of PIPs was done by consulting the ‘Opinions and decisions on PIPs and product-specific waivers’ list^3^ released on 12 November 2024. Since this document records only eight PIPs for 2024, the trend analysis is restricted to the 2007–2023 period. Although we do not have access to US PSPs data, available information on paediatric oncology drugs under development in the US has been captured through the World Health Organization (WHO) Global Observatory on Health Research and Development (R&D) (6), reporting characteristics of cancer drugs for children for the period 2007–2022 (32).^4^^,^^5^ It includes several pieces of information that are relevant to our research, such as paediatric trials for products or active substances, drug categories, phases of development, and regulatory approvals.

Information that was unavailable in the cited EMA sources was retrieved by consulting other EMA sources (i.e., https://www.ema.europa.eu/en/committees/paediatric-committee-pdco; https://www.ema.europa.eu/en/committees/committee-orphan-medicinal-products-comp), and other regulatory portals from the EMA related to human medicines, https://www.ema.europa.eu/en/medicines/download-medicine-data#medicines-69047.

Literature, pharmaceutical company websites, and clinical trials databases (e.g., clinicaltrials.gov) were consulted to complete the overall missing records.

Information on products: For each oncology paediatric MP, the following data were considered: AS classified as ‘cytotoxic chemotherapy agent’ ‘molecular targeted therapy’ or ‘immunotherapy’; indications and year of approval of the reference product, paediatric indications classified according to the WHO (33), and the European Society for Paediatric Oncology (SIOPE) (34), and year of paediatric approval.

Products were also classified as follows: (1) Drugs initially developed for adult cancers in the same indication (adult-driven products) and (2) drugs developed for a specific paediatric indication different from the initial adults’ indication (non-adult-driven products), or initially developed for children (only paediatric products).

Information on PIPs: For each agreed PIP in the oncology therapeutic areas, we analysed the following factors: the last updated decision date, the PIP indication(s), the indication(s) of the reference product when existing or of the related active substance in development, and the drug category, similarly to the analysis done for marketed products.

### 2.2 Data analysis

Analysis was conducted on collected data concerning MPs approved in the EU and the US from 2007 to 2024, as well as oncology PIPs submitted in the EU during the years covered by the Paediatric Regulation in the EU.

A descriptive analysis of the following summary data was performed: number of paediatric oncology MPs approved in the EU and in the US by year; number of PIPs granted by years of approval; number of approved paediatric oncology MPs and PIPs in each drug category: ‘cytotoxic chemotherapy,’ ‘molecular targeted therapies,’ and ‘immunotherapy’ [27]; number of paediatric oncology MPs and PIPs by paediatric therapeutic indications for cancers; number of approved paediatric oncology MPs and PIPs in each oncology indication (adult-driven indication, non-adult-driven indication including only children’s indications).

Univariate linear regression analysis was used to evaluate longitudinal trends in paediatric oncology drugs approved in the EU and the US in the period 2007–2024, and PIPs approved between 2007 and 2023. Linear regression beta coefficients were calculated to evaluate potential changes in the number of oncology drugs approved over time.

A chi-squared test was applied to compare categorical variables between the periods 2007–2017 and 2018–2024: oncology indications (adult-driven indication, non-adult-driven indication, and only-children indication); drug categories (cytotoxic chemotherapy agents, molecular targeted therapies, and immunotherapies). We adopted these two periods based on the assumption that, in 2017, the new rules were not yet consolidated but were likely first adopted by both agencies with a comparable level of application.

To compare the time trand of MPs apoved by the EMA vs the FDA according to drug category, an analysis of variance (ANOVA) was proposed.

All statistical tests were two-sided and performed using a significance (alpha) level of 0.05. Data were analysed using the Statistical Package for the Social Sciences (SPSS) software (IBM SPSS Statistics for Windows, version 29.0, IBM Corporation, Armonk, NY, United States).

## 3 Results

### 3.1 Paediatric oncology MPs in the EU and in the US

#### 3.1.1 Total approvals and trends

The number of anticancer active substances granted at least one paediatric MA in the period 2007–2024 is equal to 35 in the EU and 53 in the US. The lists of paediatric products approved in the EU vs. the US by year, evaluated in this study, are added to the Supplementary materials. Of the 35 AS marketed in the EU, 3 (*mifamurtide, temozolomide, thiotepa*) do not have an approved paediatric indication in the US, and 32 are common in both regions. Among the additional 21 ASs approved in the US, 2 have been granted a paediatric indication also in the EU in January 2025 (*avelumab* and *reprotectinib*), and 13 have an approved PIP in development. In the US, 4 molecules (*tagraxofusp-erzs*, *tazemetostat*, *tovorafenib*, and *vorasidenib*) have been classified as ‘first-in-class’ new drugs.

**Figure 1:**
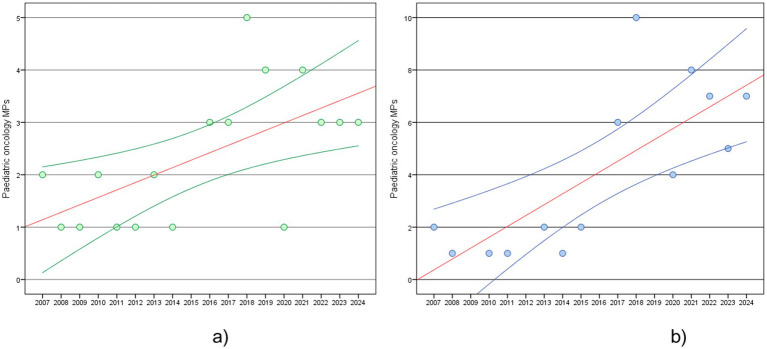
Paediatric oncology MPs, estimated regression line (red line) and 95% confidence interval (blue line): **(a)** trend in EU in 2007–2024; **(b)** trend in US in 2007–2024.

The trends in paediatric oncology MPs approvals are examined over time (2007–2024) in the EU and the US (Figure 1). Based on regression analyses, the frequency of paediatric oncology MPs in the EU shows a significant increase over time (*p* = 0.009), with an average growth of 0.14 MPs per year. This suggests a steady but relatively slow increase in approvals. Instead, the US experienced a significant rise in approvals (*p* = 0.002), occurring at a rate of 0.42 MPs per year, reflecting a quicker approval pace than the EU.

#### 3.1.2 Therapeutic indications

Table 1 shows the paediatric oncology indications related to the approved products distributed according to the main therapeutic categories: haematology (HMs), solid tumours (STs), and supportive. Collectively, paediatric indications account for 49 in the EU and 79 in the US. Across both regions, 62 paediatric indications appear in product labels.

**Table 1:** Classification of approved paediatric oncology indications by the EMA and FDA according to tumour type.

Main therapeutic categories	Paediatric tumour type	EU-approved N (%)	US-approved N (%)	Notes^1^
Haematology Total EU: 46.9% Total US: 46.8%	leukaemias	17 (34.7)	27 (34.2)	
	lymphomas	6 (12.2)	10 (12.7)	
Solid tumours Total EU: 42.9% Total US: 44.3%	solid tumors multiple indication	2 (4.1)	10 (12.7)	*Repotrectinib* approved in the EU in January 2025
	neuroblastomas	1 (2.0)	3 (3.8)	*Eflornithine* recently submitted for marketing authorisation in the EU
	brain tumor	6 (12.2)	7 (8.9)	
	osteosarcomas and Ewing’s sarcomas	3 (6.1)	2 (2.5)	*Mifarmurtide* rejected in the US
	melanoma	3 (6,1)	3 (3,8)	
	tyroid cancer colonrectal carcinoma, other^2^	6 (12.2)	10 (12,7)	*Avelumab* approved in the EU in January 2025
Supportive Total EU: 10.2% Total US: 5.1%		5 (10.2)	4 (5.1)	
	Total	49	79	EU/US total 82

1 The “Notes” refers to additional information argumented in the discussion.

2 E.g. Merkel carcinoma.

#### 3.1.3 Adults-driven and non-adults-driven paediatric oncology indications

In Table 2, approved indications are classified as adult-driven or non-adult-driven. The percentage of non-adult-driven indications increases significantly in the EU (*p* = 0.024) after 2018, with no difference found when comparing the EU and US approvals (*p* = 0.767).

**Table 2:** Classification according to indication category.

Period	EMA N (%)			FDA N (%)		
	Adult-driven indication	Non-adult-driven indication	TOTAL	Adult-driven indication	Non-adult-driven indication	Total
2007–2017	21 (95.5)	1 (4.5)	22	17 (85.0)	3 (15.0)	20
2018–2024	19 (70.4)	8 (29.6)^1^	27	48 (81.4)	11 (18.6)^1^	59
2007–2024	40 (81.6)	9 (18.4)	49	65 (82.3)	14 (17.7)	79
Between periods *p*-value	0.024^2^		-	0.712^3^		-
EMA vs. FDA *p*-value	0.767^4^					

1 Of which 5 are ‘only paediatric’.

2 The chi-square statistic is 5.087.

3 The chi-square statistic is 0.136.

4 The chi-square statistic is 0.088.

Of note, 5 out of 9 non-adult-driven indications in the EU (*dinutuximab* (*beta*), *clofarabine*, *selumetinib*, *sodium thiosulfate*, and *trametinib dimethyl sulfoxide*) and 5 out of 12 in the US (*dinutuximab* (*beta*), *clofarabine*, *selumetinib*, *calaspargase pegol*, and *tovorafenib*) are authorised as ‘only paediatric’.

#### 3.1.4 Drug category of paediatric oncology products

The distribution by drug category is reported in Table 3, demonstrating that in the EU, 16 (45.7%) of 35 drugs belong to the groups of ‘targeted drugs’ and 9 (25.7%) to ‘immunotherapy’ vs. 8 (22.9%) classified as ‘cytotoxic chemotherapy’. In the US, 24 (45.3%) of 53 are targeted drugs, 14 (26.4%) are immunotherapy, and 10 (18.9%) are cytotoxic chemotherapy. CAR-T cell therapy is approved in both regions for the treatment of refractory or relapsed B-cell acute lymphoblastic leukaemia in post-transplant treatment settings.

**Table 3:** Classification of MPs approved by EMA and FDA according to drug category.

Period	Drug category: EMA N (%)				Tot	Drug category: FDA N (%)				Tot
	Targeted	Immuno	Chemot	other		Targeted	Immuno	Chemot	other	
2007–2017	4 (28.6)	4 (28.6)	5 (35.7)	1	14	5 (33.3)	5 (33.3)	4 (26.7)	1	15
2018–2024	12 (57.1)	5 (23.8)	3 (14.3)	1	21	19 (50.0)	9 (23.7)	6 (15.8)	4	38
2007–2024	16 (45.7)	9 (25.7)	8 (22.9)	2	35	24 (45.3)	14 (26.4)	10 (18.9)	5	53

Time-trend analysis. targeted drugs *p*-value = 0.254. immunotherapy drugs *p*-value = 0.363. cytotoxic chemotherapy drugs *p*-value = 0.455.

A total of 62 paediatric indications were collectively recorded (see subsection 3.1.2), with 93 and 65% attributed to targeted or immunologic agents for STs and HMs, respectively (*p* = 0.013). This indicates an advantage for ST in terms of innovative drug categories.

In the US, approvals of immunotherapy agents increased more compared to the EU. Among immunoagents, five approved drugs are tissue-agnostic therapies (*larotrectinib*, *pembrolizumab*, *entrectinib*, *selpercatinib*, *dabrafenib/trametinib in combination*), and two agents are epigenetic factors. In particular, two targeted drugs (*larotrectinib* and *entrectinib*) have received an indication for paediatric extracranial solid tumours that display a Neurotrophic Tyrosine Receptor Kinase (NTRK) in the EU and in the US for children from 1 month and over, thus also covering neuroblastomas.

A shift was recorded in the two timeframes before and after 2018 in the increasing approval of targeted and immunoagents over chemotherapies in both the US and EU. However, no significant shifts were noticed between the two regions, for the whole sample and for the three drug categories.

### 3.2 Paediatric oncology PIPs in the EU

A total of 216 PIPs corresponding to 172 new AS or expanded indications of authorised MPs were analysed. It is important to note that the number of recorded PIPs in the EMA database in 2024 is limited. Thus, to prevent bias due to the small sample size, data from 2024 were excluded from the trend analysis.

**Figure 2:**
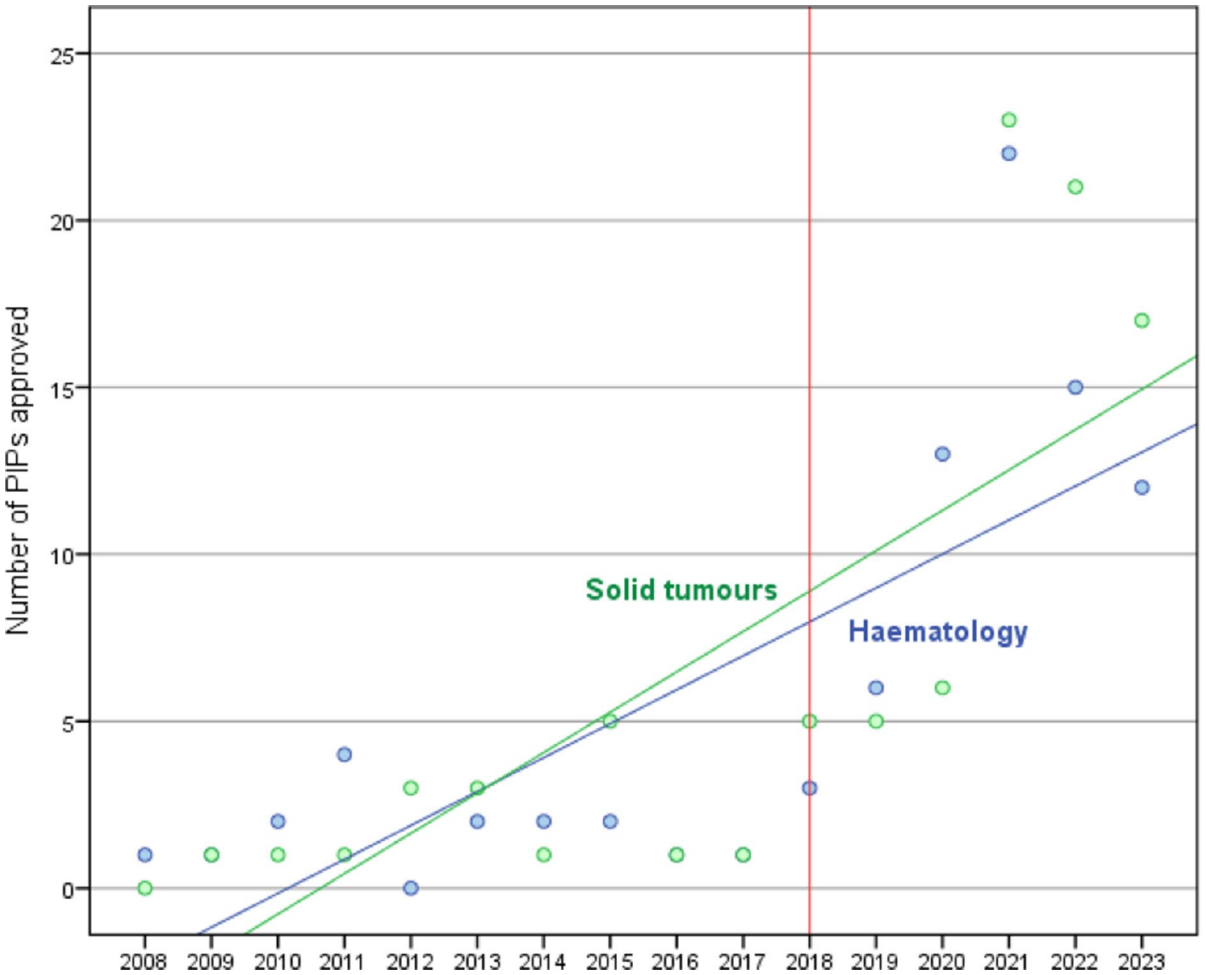
Trend of PIPs approved between 2008 and 2023 according to tumour type classification: solid tumours (green line) and haematology (blue line).

The trends in the approval of PIPs for paediatric oncology MPs in the EU in the periods 2008–2017 and 2018–2023 (Figure 2) show a significant (*p* < 0.001) linear relationship (R^2^ = 0.629) between PIPs decisions and time (years), with an average increase of 2.6 PIPs decisions each year (2008–2023). When comparing the two periods, an average annual increase of 0.3 PIPs (*p* = 0.211) is observed from 2008 to 2017. In contrast, the period from 2018 to 2023 experienced a more significant increase, with an average of 7.1 PIPs (linear regression model *p*-value < 0.001) approved each year.

In Table 4, we report the paediatric oncology indications related to the 216 approved PIPs according to the primary tumour type.

**Table 4:** Classification of PIPs according to tumour type.

Main therapeutic categories	Paediatric tumour type	PIPs N
Haematology Total 98 (45%)	leukaemias	57
	lymphomas	41
Solid tumor Total 111 (51%)	solid tumor multiple indications	53
	brain tumors	17
	osteosarcomas and Ewing’s sarcomas	5
	neuroblastomas	7
	melanoma	13
	other (rabdomiosarcoma and tyroid, colon and breast cancers, etc.)	16
Supportive (3%)	conditioning, toxicity, post-trasplant (etc.)	7
	Total	216

Notably, we observed a relevant increase in PIPs for STs, which currently surpass those of HM PIPs. While the majority of the ST PIPs are for multiple paediatric indications, several PIP indications address currently uncovered paediatric tumours, such as brain tumours. In contrast, for bone tumours and neuroblastomas, the agreed PIPs are still limited.

In Table 5, PIPs are classified into adult-driven and non-adult-driven, which also include only-children indications. A significant difference exists between the two study periods (*p* = 0.003). The 14 (34.1%) PIPs approved for non-adult-driven and only-children indications before 2018 increased to 84 (60.0%) in the period 2018–2024.

**Table 5:** Classification of PIPs according to indication categories.

Period	EMA-PIP decision N (%)				
	Adult-driven-indication	Non-adult-driven indication	Only children indication	Total	*p-*value
2007–2017	27 (65.9)	14 (34.1)	0 (0.0)	41	0.003^1^
2018–2024	56 (40.0)	79 (56.4)	5 (3.6)	140	
Total	83 (45.9)	93 (51.4)	5 (2.8)	181	-

1. The chi-square statistic is 8.537.

PIPs were also classified by drug categories. A significant difference (*p*-value < 0.001) exists between the two study periods. Before 2018, 6 (18.8%) PIPs were approved for targeted molecules, and 5 (15.6%) for immune-related molecules, while after 2018, approvals rose to 55 (39.3%) for targeted molecules and 41 (29.3%) for immunoagents. A total of 15 of 30 PIPs classified as ‘other’ in the table are gene therapy, including CAR-T cell therapy. Of these, 5 are for solid cancers and 10 for haematological malignancies.

## 4 Discussion

The results from this study suggest that in recent years, the panorama of paediatric oncology therapies has significantly changed. Modifications are both quantitative (more active agents on the market and more indications covered) and qualitative, as innovative drugs prevail over cytotoxic chemotherapeutic agents, and medicines developed to meet specific paediatric needs have increased. Peculiar points to be discussed in this study are detailed below.

### 4.1 Paediatric oncology products are increasing both in the EU and in the US, but at a different pace

Our results show that an increasing trend of approved oncology drugs is present both in the EU and in the US. However, while up to 2017, a total of approximately 1.5 new drugs per year were approved in both regions, in the subsequent period, the approval rate has become significantly higher in the US than in the EU, with 5.4 new products/year for the former vs. 3 products/year for the latter. This resulted in a notable discrepancy, with 35 new oncology drugs and 49 new paediatric indications approved in the EU, compared to 53 drugs and 79 new indications in the US.

Despite the existing gap, the two paediatric markets are developing in parallel. A total of 32 marketed products of 35 in the EU have also been marketed in the US. Two additional products received marketing approval in 2025 (*avelumab* and *reprotectinib*, a targeted therapy that received conditional authorisation).^6^ A further reduction of the gap is expected by the completion of some MAs that are ongoing (i.e., eflormithine, receiving FDA approval in 2023 to reduce the risk of relapse in adult and paediatric high-risk neuroblastoma). Additionally, we found that of the 21 MPs authorised only in the US, 13 have an ongoing PIPs.

Two marketed products in the EU have been refused (*mifamurtide*) or never approved (*temozolamide*) in the US, but for the majority, the two Agencies have released very similar opinions, even if with a systematic delay in the EU compared to the US. The approved indications for products authorised both in the EU and in the US slightly differ, resulting in more paediatric indications/age groups being covered by the available treatments in the US.

### 4.2 The approved indications show an increasing trend for products covering specific paediatric needs

Regarding the approved indications, the most frequent paediatric indications approved for these drugs, in both countries, were for leukaemias, the most common paediatric cancer and still the leading cause of death among the paediatric population (25). Globally, haematological malignancies (HMs) account for a total of 47%, while in both regions there is an increase in new paediatric drugs approved for STs (47.7% in the EU and 48.9% in the US; Table 1). In fact, while the spectrum of tumour types that occur in children is significantly different from that observed in adults, historically, paediatric ST drugs have been given little consideration during the development process of adult drugs (35). Considering that STs account for 60% of all paediatric malignant neoplasms, this increase represents a relevant, yet insufficient, novelty in the sector.

Furthermore, despite unmet needs for new treatments in specific areas (24, 36), such as osteosarcomas, Ewing’s sarcoma, retinoblastoma AND Wilms tumours, STILL REMAIN IN BOTH REGIONS AND mainly in the EU, unexpectedly, our analysis shows an increase in approved indications for cancer that are very rare in children, including melanoma, thyroid cancer, and other indications previously lacking any treatment [e.g., plexiform neurofibromas (PN) with neurofibromatosis type 1 (NF1); myofibroblastic tumour (IMT)] (37).

In this context, differences between the EU and the US appear to be of great relevance. Those refer to the number of approved drugs; for example, *dinutuximab* is the only MP approved for high-risk neuroblastoma in the EU, while two new drugs, *iobenguane I-131* and eflornithine, are approved in the US. Similarly, two drugs are approved for solid tumours with multiple indications in the EU compared to a total of 10 drugs approved in the US. Several drugs (*pembrolizumab*, *selpercatinib*, *nivolumab*, *relatlimab*, and *trametinib dimethyl sulfoxide*) are marketed in the EU for different indications or are approved only for adults.

Moreover, three of four ‘first-in-class’ molecules approved in the US (of which two are for the treatment of malignant glioma) have never been approved in the EU—either for adults or for children.

These data demonstrate that in recent years a new trend exists that may mitigate the hypothesis at the basis of the paediatric legislation revision in the EU, which has substantially failed (28, 29), and is in support of the RACE initiative in the US. It consists of the increase in the 2018—2024 period, of the percentage of non-adult-driven indications, both in the EU and in the US. Moreover, in five cases in each region, an ‘only-children’ indication, entirely independent of any interest driven by the adult drugs market, was approved.

### 4.3 New molecularly targeted and immunologic drugs approved for paediatric use are on the rise

We believe that the real revolution driving new trends and substantial growth on the paediatric oncology market is the rise of innovative molecular targeted and immunologic targeted drugs (17). After years in which chemotherapies have represented the prevalent or only therapeutic option in paediatric oncology, these groundbreaking therapies are now prevailing (38). As shown in our study, in the period 2017–2024, among the approved paediatric drugs, only 8 of 35 in the EU and 10 of 53 in the US were chemotherapeutic agents. On the contrary, a total of 27 and 43 drugs, respectively, in the EU and in the US are derived from more promising innovative technologies.

Unfortunately, several innovative drugs are only approved in the US, such as eight molecularly targeted drugs (*bosutinib, cabozantinib, ibrutinib, pralsetinib, revumenib, tovorafenib, vorasidenib*, and *repotrectinib*) and eight immune and other biological agents (*atezolizumab*, *avelumab*, *brentuximab vedotin, eflornithine*, *emapalumab-lzsg*, *inotuzumab ozogamicin*, *iobenguane i-131*, and *tagraxofusp-erzs*).

However, based on the information available today, we can affirm that incorporation of targeted agents into upfront treatment regimens is leading to incremental improvements in event-free survival for many children (39). In particular, for rare or challenging-to-treat cancers, the increasing feasibility of molecular profiling has provided specific treatment options to patients with some of the greatest needs (40). Several ongoing study protocols and trials are demonstrating ameliorated tolerability and ameliorated safety profile (i.e., binatumumab in high-risk acute lymphoblastic leukaemia in infant) (41); decreased toxicity due to reduction of chemotherapy or, possibly, avoidance of radiotherapy in selected cases (i.e., nivolumab or brentuximab) (42); positive result also in monotherapy (i.e., selpercatinib in the treatment of refractory solid tumours with RET mutations (43); superior clinical outcomes compared to historical data reported from Paediatric Oncology Networks in poor prognosis paediatric malignancies (i.e., pediatric high-grade glioma) (44). In conclusion, the clinical impact of these molecules and their contribution to better survival and quality of life (QoL) appears promising. Benefits are expected to increase, especially if the use of innovative drugs expands in clinical practice beyond the experimental use in restricted experimental populations.

A clearer understanding of the clinical relevance of these advancements will need time and further evaluation, also considering that for all these drugs, the clinical evidence at the time of drug approval may be limited, both for the limited time from approval and, often, due to the expedited MA procedures adopted by the Agencies. These procedures, granted to facilitate quicker access to promising therapies, in fact, shorten the time for the evaluation and require less data than typically deemed necessary (45).

### 4.4 Real-word access

Despite the unprecedented speed of innovation in paediatric oncology that has occurred in the last few years, profound disparities still exist for patient access to cancer therapies, due to various reasons, including geographic- and health systems-related issues.

It is now well recognised that there is a shortage and limited access to oncology drugs in low- and middle-income countries, primarily due to poverty, scarcity of resources, and inadequate investments in health and research, including essential medicines listed by the WHO (46, 47). However, unequal access to anticancer medicines for children and adolescents is a reality also in Europe. This has also been recently demonstrated by a large survey among several EU countries, which shows that medicines defined as essential were available for the treatment of childhood and adolescent cancer continuously and across Europe, albeit in only a limited percentage (63%), with substantial differences in different countries (48). These differences cannot be explained by socioeconomic inequalities only, while public health policies are relevant (49). In particular, in Europe, the existing differences among the price and reimbursement procedures in each MS have a huge impact on the concrete usability of the drugs and the will of the Marketing Authorisation Holder (MAH) to deliver the drug in each EU-MS (16). For instance, in the post-marketing phase, the drugs often continue to be used mainly in the Academic Reference Centre and according to compassionate use programmes. As an example, in a ‘one centre’ investigation, conducted up to February 2025 (50) at the Paediatric Oncology reference centre of the University of Bari, it has been demonstrated that only 50% (i.e., 17) of innovative drugs approved in the EU are fully available for local use.

### 4.5 Several PIPs in development in the EU show similar trends as approved products

All the trends already described for paediatric oncology approved products have also been confirmed through PIPs analysis, demonstrating a significant annual increase occurred after 2018, when the class waiver revision entered fully came into force, the number of approved PIPs each year expanded from 0.1 per year to 7 per year, and the rate of PIPs associated with targeted therapies and immunotherapies doubled after 2018.

Notably, we observed a relevant increase in PIPs for solid tumors with 50.6%, compared to 44.7% for HM PIPs. The majority of the ST PIPs are for multiple paediatric indications. Several other PIP indications are for tumours for which new products are lacking, such as brain tumours and other rare tumours. Unfortunately, bone tumours and neuroblastoma are still underrepresented among approved PIPs. As for marketed drugs, several PIPs are approved for melanoma and other rare paediatric indications.

More interestingly, in this timeframe, 84 (60.0%) PIPs have been granted approval for non-adult-driven indications (Table 5), prevailing over the 56 (40.0%) PIPs approved for adult-driven ones. In addition, the increasing number of PIPs approved to develop only-paediatric indications is very valuable, considering that these PIPs are not mandatory but the sponsors voluntarily chose to submit them and thereby received the PDCO regulatory opinion and other useful available support from the EMA (e.g., presubmission meetings, scientific advice, paediatric incentives, etc.).

Notwithstanding the comparability of datasets between the US and the EU is limited due to the lack of comparable sources of data on PIP and PSP, information on paediatric oncology drugs on development captured through the WHO Global Observatory on Health R&D (6) covering the period 2007—2022, allowed us to demonstrate that gliomas, neuroblastoma, and osteosarcoma are the top three malignancies with the highest number of drugs in clinical trials. While few trials are ongoing covering multiple indications, gliomas, neuroblastoma, and osteosarcoma are the top three malignancies with the highest number of drugs in clinical trials, and the more frequent types of drugs are vaccines mainly covering glioma, neuroblastoma, and medulloblastoma (51).

### 4.6 The persistent European gap in light of the new pharmaceutical strategy

In Europe, the advancements achieved up to today demonstrate that the current Paediatric Regulation, which includes a scientific Paediatric Committee and a clear obligation to develop *ad hoc* paediatric medicinal products, has positively acted in progressing the paediatric medicines status and availability (Table 6).

**Table 6:** Classification of PIPs according to drug category (Immuno, immunotherapy; chemo, chemotherapy).

Period	Drug category: PIPs					
	Targeted	Immuno	Chemo	other	total	*p-value*
2007–2017	6 (18.8)	5 (15.6)	14 (43.8)	7 (21.9)	32	< 0.001^1^
2018–2024	55 (39.3)	41 (29.3)	21 (15.0)	23 (16.4)	140	
2007–2024	61 (35.5)	46 (26.7)	35 (20.3)	30^2^ (17.4)	172	-

1. The chi-square statistic is 16.078.

More specifically, in the EU, the revision of class waivers adopted by the Paediatric Committee (PDCO) in 2015 was not oriented to introduce a similar MoA approach directly. However, it emerges that, in parallel with the US, also the EMA-PDCO has voluntarily adopted an MoA approach in its paediatric drugs evaluation process, even if this approach was not stated in the Paediatric legislation. As shown above, for the effects of the Paediatric Regulation in the EU, we observed an increase in oncology drugs for several new paediatric indications, focused on paediatric diseases instead of on adult-driven indications. This aspect demonstrates, within others, that in contrast with the current EU position on the new Pharmaceutical Legislation - claiming for the PDCO abolition due to the effects of the Paediatric Regulation repealing—the EU-PDCO scientific and regulatory approach, remains valid, with the current Regulation successfully incorporating several relevant scientific advancements. However, our data also demonstrated that there are relevant disparities between the US and the EU in terms of the number and quality of paediatric oncology drugs. We suggest that the advancements demonstrated in the US are the result of significant regulatory modifications initiated by the 2017 FDA Reauthorization Act (FDARA) revision, further reinforced by the incorporation of the RACE Act. Notably, the RACE Act not only requires developing a PSP for molecules with an MoA effective for paediatric tumours, and eliminates the automatic waiver for rare oncological drugs, but also incentivises the prioritisation of novel paediatric anticancer drugs by providing incentives and support to the MAHs (19). All these measures are in line with the different legislative and regulatory approaches in the US (52) that appear more effective in promoting paediatric medicines development than in the EU.

In the EU, at the current status of debate, waiting for the final approval of the new pharmaceutical Directive and Regulation, certain advantages are expected for children, that is, the formal introduction of the Mechanism of Action (MoA) criterion in the PIP approval process and some simplification in the Paediatric Investigational Plan (PIP) application/approvals procedures. While some concerns have been expressed regarding the lack of specific solutions covering other relevant topics (e.g., dedicated funds promoting paediatric research, measures to support the small paediatric medicines market, identification of the paediatric unmet therapeutic needs as specific, etc., all of these to be taken specifically in consideration in order to promote supportive measures in critical area such as oncology) (30, 31, 53).

In particular, several concerns have been expressed with reference to the repealing of the Paediatric Regulation, while the inclusion of several measures formerly part of the Paediatric Regulation, into the new proposed generalist Regulation and Directive, seems to make it very difficult to appreciate if these solutions will be adequate to solve discrepancies and limitations in the paediatric field.

## 5 Conclusion

After years, it appears that the paediatric oncology framework is progressing in the EU as it is in the US, with more products, several newly approved paediatric indications, and a large number of paediatric developmental plans in place. Innovative drugs now prevail over chemotherapeutic products, with expected reduced toxicity and increased tumour-targeted specificity. Drugs for solid tumours, covering paediatric tumour indications, particularly benefit from these progresses, and PIPs for these indications now prevail over drugs for haematological malignancies.

The EU and the US are clearly working in parallel on these efforts, but a relevant gap still exists.

Unlike the EU, the US has proven to be at the forefront with the approval of the latest therapies, and the enactment of the RACE Act appears to have shortened paediatric cancer drug development times by 3 years compared to the pre-RACE period (54, 55).

Addressing this discrepancy in the right way should represent an urgency for Europe. We recommend not wasting the opportunity offered by the ongoing reform of the pharmaceutical legislation. However, considering that this reform proposes the repeal of the Paediatric Regulation, there is a risk that, without other initiatives and substantial investments in paediatric care, no substantial progress may be expected in the near future to address the several therapeutic unmet needs of children.

### 5.1 Limitations

This study is based on data collected from public repositories, further incorporated into a structured paediatric medicines database (EPMD). Difficulties were encountered in: (a) completing data collection from the EU (i.e., with reference to the EMA dataset on PIPs and waivers, which showed few updates in 2023 and 2024); (b) performing an analysis of US paeditric developmental plans due to the absence of a PSP database; and (c) heterogeneity in data sources between the two countries.

## Data Availability

The original contributions presented in the study are included in the article/Supplementary material, further inquiries can be directed to the corresponding author.

## Footnotes

^1^ https://www.teddynetwork.net/european-paediatric-medicines-database-epmd/

^2^ https://www.ema.europa.eu/en/medicines/download-medicine-data#medicines-69047

^3^ https://www.ema.europa.eu/en/medicines/download-medicine-data#paediatric-investigation-plans-69049

^4^ https://www.who.int/observatories/global-observatory-on-health-research-and-development/monitoring/pediatric-cancer-drug-pipeline-characteristics

^5^WHO Observatory on Health Research is a validated source that primarily draws its validation sources from a combination of existing data, reports, and new information gathered from various sources, including WHO’s own data and reports, as well as external data providers. By combining these sources and validation methods, the observatory plays a crucial role in monitoring and analysing R&D related to paediatric oncology clinical trials.

^6^ https://www.esmo.org/oncology-news/ema-recommends-granting-a-conditional-marketing-authorisation-for-repotrectinib
