# Supplementary material for: Scientific and regulatory progress in advancing paediatric oncology drug development in the EU and in the US
Source: Front Med (Lausanne). 2025 Sep 26;12:1642279. doi: 10.3389/fmed.2025.1642279 (PMC12511141; doi:10.3389/fmed.2025.1642279)
Supplement: Supplementary file 1 [file Data_Sheet_1.PDF]

MA	Medicinal Product (MP)	Active Substance	Category	Category #1	Adults and Paediatric indication	Paediatric indication different from adults or only paediatric	Paediatric indication approval year in EU	Paediatric indication approval year in US
EU/US	Atriance (arranon)	nelarabine	chemotherapy	chemotherapy	1- T-cell acute lymphoblastic leukaemia (T-ALL)		2007	2005
EU/US	Atriance (arranon)	nelarabine	chemotherapy	chemotherapy	2- T-cell lymphoblastic lymphoma (T-LBL) not responded to or relapsed		2007	2005
EU/US	Atriance (arranon)	nelarabine	chemotherapy	chemotherapy	3-anaplastic astrocytoma in progression		2007	2005
EU	Mepact	mifamurtide	immunomodulator	immunotherapy	1- high-grade resectable non-metastatic osteosarcoma after macroscopically complete surgical resection		2009	
EU/US	Votubia (Afinitor)	everolimus	Target therapy Kinase Inhibitor and mTOR Inhibitor	target therapy	1- Subependymal giant cell astrocytoma (SEGA) associated with TSC		2011	2010
EU/US	Xaluprine (PURINETHOL)	mercaptopurine monohydrate	chemotherapy	chemotherapy	1- treatment of acute lymphoblastic leukaemia (ALL)		2012	2014
EU/US	GLIVEC	imatinib	Targeted therapy in the form of selective tyrosine kinase inhibitors (TKIs)	target therapy	1-Philadelphia chromosome positive chronic myeloid leukaemia (CML) (Ph+ CML)		2013	2006
EU/US	GLIVEC	imatinib	Targeted therapy in the form of selective tyrosine kinase inhibitors (TKIs)	target therapy	2-newly diagnosed Philadelphia chromosome positive acute lymphoblastic leukaemia (Ph+ ALL)		2013	2013
EU/US	Caprelsa	vandetanib	small molecule tyrosine kinase inhibitor	target therapy	1- aggressive and symptomatic medullary thyroid cancer		2016	2011
EU/US	Spectrila (RYLAZE)	asparaginase	chemotherapy	chemotherapy	1- Combination therapy for the treatment of acute lymphoblastic leukaemia (ALL)		2016	2021
EU/US	Oncaspar	pegaspargase	chemotherapy	chemotherapy	1- antineoplastic combination therapy in acute lymphoblastic leukaemia (ALL) with ipersensitivity to E.C.		2016	2006
EU/US	Qarziba (previously Dinutuximab beta EUSA and Dinutuximab beta Apeiron)	dinutuximab (beta)	immuno. via the antibody-dependent cellular cytotoxicity (ADCC)	immunotherapy		1-treatment of high-risk neuroblastoma in patients aged 12 months and above,	2017	2015
US	Yervoy	ipilimumab	immuno.CTLA-4 monoclonal antibody	immunotherapy	2-metastatic colorectal cancer (mCRC) that has progressed			2018
EU/US	Yervoy	ipilimumab	immuno.CTLA-4 monoclonal antibody	immunotherapy	1- treatment of advanced (unresectable or metastatic) melanoma		2018	2023
EU/US	Blincyto	blinatumomab	immuno. bispecific T-cell engager	immunotherapy	1- monotherapy for the treatment of Philadelphia chromosome negative CD19 positive B-precursor ALL (in first or second and successive line)		2021	2018

MA	Medicinal Product (MP)	Active Substance	Category	Category #1	Adults and Paediatric indication	Paediatric indication different from adults or only paediatric	Paediatric indication approval year in EU	Paediatric indication approval year in US
EU/US	Mylotarg	gemtuzumab ozogamicin	1-CD33 targeting and calicheamicin-induced DNA damage	immunotherapy	1- combination therapy with daunorubicin (DNR) and cytarabine (AraC) for the treatment of patients age 15 years and above with previously untreated, de novo CD33-positive acute myeloid leukaemia (AML)		2018	2017
EU/US	Kymriah	tisagenlecleucel	1-autologous, murine anti-CD19 Chimeric Antigen Receptor T cell (CAR-T) a targeted immune-modulating therapy	target therapy		1-B-cell acute lymphoblastic leukaemia (ALL) that is refractory, in relapse post-transplant or in second or later relapse.	2018	2017
EU/US	Mozobil	Plerixafor	acting on CD34+ cells	target therapy	1- in combination with granulocyte-colony-stimulating factor for cells collection and subsequent autologous transplantation		2019	2008
EU/US	Vitrakvi	Larotrectinib	1. targeted therapy drugs called cancer growth inhibitors (NTRK gene +)	target therapy	1- advanced solid tumours that display a Neurotrophic Tyrosine Receptor Kinase (NTRK) gene fusion		2019	2018
EU/US	Pedmarqsi (Pedmark)	Sodium thiosulfate	other	other		Prevention of platinum-induced ototoxic hearing loss	2023	2022
EU/US	Ivozall (Clolar)	clofarabine	cytotoxic	chemotherapy		1- acute lymphoblastic leukaemia (ALL) in paediatric patients who have relapsed or are refractory after receiving at least two prior regimens	2019	2004
EU/US	Rozlytrek	Entrectinib	immunoterapyoral tyrosine kinase inhibitor	immunotherapy	1- Treatment of solid tumours expressing a neurotrophic tyrosine receptor kinase (NTRK) gene fusion		2020	2023
EU/US	Blincyto	blinatumomab	immunotherapy (bispecific T-cell engager)	immunotherapy	2- high-risk first relapsed Philadelphia chromosome negative CD19 positive B-precursor ALL as part of the consolidation therapy:;		2018	2017
EU/US	Blincyto	blinatumomab	immunotherapy (bispecific T-cell engager)	immunotherapy	3-relapsed or refractory CD19-positive B-cell precursor acute lymphoblastic leukemia (ALL) in adult and pediatric patients (US);		2018	2018
EU/US	Blincyto	blinatumomab	immunotherapy (bispecific T-cell engager)	immunotherapy	4-treatment of CD19-positive B-cell precursor acute lymphoblastic leukemia (ALL) in first or second complete remission with minimal residual disease (MRD) greater than or equal to 0.1% (US)		2018	2024
EU/US	Keytruda	Pembrolizumab	immunotherapy	immunotherapy	3- relapsed or refractory classical Hodgkin lymphoma melanoma unresectable, melanoma IIb and IIc		2021	2017 - 2018 - 2020 - 2021
EU/US	Retsevmo	Selpercatinib	1 inhibition of mutated forms of RET tyrosine kinases	target therapy	1-advanced RET-mutant medullary thyroid cancer (MTC)		2021	2024
EU/US	Koselugo	Selumetinib	1-selectively inhibiting MEK1 and MEK2	target therapy		1-Treatment of symptomatic, inoperable plexiform neurofibromas (PN) with neurofibromatosis type 1 (NF1)	2021	2020

MA	Medicinal Product (MP)	Active Substance	Category	Category #1	Adults and Paediatric indication	Paediatric indication different from adults or only paediatric	Paediatric indication approval year in EU	Paediatric indication approval year in US
EU/US	Xalkori	Crizotinib	1tyrosine kinase receptor inhibitor that targets several tumours targets	target therapy		1- treatment of systemic anaplastic lymphoma kinase (ALK)-2 positive anaplastic large cell lymphoma (ALCL) -	2022	2021
EU/US	Xalkori	Crizotinib	1 kinase inhibitors	target therapy		2- unresectable inflammatory myofibroblastic tumour (IMT)	2022	2022
EU/US	Opdualag	nivolumab, Relatlimab	target	target therapy	1- first line treatment of advanced (unresectable or metastatic) melanoma with tumour cell PD L1 expression < 1%; 2- in combination		2022	2022
EU/US	Opdivo	Nivolumab	target	target therapy	1-in combination with nivolumab in metastatic colorectal cancer (mCRC) that has progressed		2015	2018
EU/US	Enrylaze	crisantaspase	chemotherapy	chemotherapy	1- component of a multi-agent chemotherapeutic regimen for the treatment of acute lymphoblastic leukaemia (ALL) and lymphoblastic lymphoma (LBL)		2023	2021
EU/US	Finlee<br>(Tafinlar e Mekinist)	dabrafenib	targeted immune-modulating therapy	target therapy	1-low-grade glioma (LGG) with a BRAF V600E mutation		2023	2023
EU/US	Trecondi<br>(Grafapex)	Treosulfan	chemotherapy	chemotherapy	1- conditioning treatment prior to allogeneic haematopoietic stem cell transplantation after malignant and non-malignant diseases.		2019	2025
EU/US	Spexotras	Trametinib dimethyl sulfoxide	Target therapy to inhibit the activity of MEK1 and MEK2	target therapy		1.low-grade glioma (LGG) with a BRAF V600E m<br>2.high-grade glioma (HGG) with a BRAF V600E m	2024	2021
EU/US	RATIOGRASTIM	filgrastim	other	other	1-Reduction in the duration of neutropenia and the incidence of febrile neutropenia in patients treated with chemotherapy for malignancy		2008	2015
EU	TEMODAL	temozolomide	chemotherapy	chemotherapy	1-recurrent or progressive glioblastoma multiforme		2010	
EU/US	Xgeva	Denosumab	monoclonal antibody that binds the cytokine RANKL	immunotherapy	1-Treatment of giant cell tumour of bone that is unresectable or where surgical resection is likely to result in severe morbidity.		2014	2013
EU/US	MABTHERA<br>Ruxience - Rixathon - Blitzima - Riximyo - Truxima	Rituximab	targeting CD20,	target therapy	1- untreated advanced stage CD20 positive diffuse large B-cell lymphoma (DLBCL),2-Burkitt lymphoma (BL)/Burkitt leukaemia (mature B-cell acute leukaemia) (BAL) 3-or Burkitt-like lymphoma (BLL).		2017	2021
EU/US	Tasigna	nilotinib	target therapy inhibits the tyrosine kinase activity of the BCR-ABL protein	target therapy	1- newly diagnosed Philadelphia chromosome positive chronic myelogenous leukaemia (CML) in the chronic phase,		2017	2018
EU/US	Tasigna	nilotinib	target	target therapy	2- chronic phase Philadelphia chromosome positive CML with resistance or intolerance to prior therapy including imatinib.		2024	2018

MA	Medicinal Product (MP)	Active Substance	Category	Category #1	Adults and Paediatric indication	Paediatric indication different from adults or only paediatric	Paediatric indication approval year in EU	Paediatric indication approval year in US
EU/US	Sprycel	dasatinib	target t. ATP-competitive protein tyrosine kinase inhibitor (	target therapy	1- (Ph+ CML-CP and Ph+ ALL) leukemia		2018	2017
EU/US	Sprycel	dasatinib	target	target therapy	2- Ph+ CML-CP resistant or intolerant to prior therapy including imatinib.		2024	2018
EU	Tepadina	thiotepa	chemotherapy	chemotherapy	1- conditioning treatment prior to allogeneic or autologous haematopoietic progenitor cell transplantation (HPCT) in haematological diseases		2010	
US	Tecentriq	Atezolizumab	engineered monoclonal antibody of IgG1	immunotherapy	1-As a single agent, for the treatment of adult and pediatric patients 2 years of age and older with unresectable or metastatic alveolar soft part sarcoma (ASPS)			2022
US	Bavencio	Avelumab	monoclonal antibody . The drug targets the PD1 receptor, an immune checkpoint.	immunotherapy	1-Treatment of adults and pediatric patients 12 years and older with metastatic Merkel cell carcinoma (MCC)			2017
US	Vidaza	Azacitidine	epigenetic	other		Treatment of pediatric patients aged 1 month and older with newly diagnosed Juvenile Myelomonocytic Leukemia (JMML)		2022
US	bosulif	Bosutinib	Target therapy. protein tyrosine kinase inhibitor (TKI)	target therapy	adult and pediatric patients 1 year of age and older with chronic phase Ph+ intolerant to prior therapy. (1)			2023
US	Adcetris	Brentuximab vedotin	antibody-drug conjugate	immunotherapy		pediatric patients 2 years and older with previously untreated high risk classical Hodgkin lymphoma (cHL), in combination		2022
US	Cabometyx	Cabozantinib	small molecule tyrosine kinase inhibitor	target therapy	1-adult and pediatric patients 12 years of age and older with locally advanced or metastatic differentiated thyroid cancer (DTC)			2021
US	Asparlas	Calaspargase Pegol	chemotherapy	chemotherapy		acute lymphoblastic leukemia in pediatric and young adult patients age 1 month to 21 years		2018
US	Vyxeos	Daunorubicin and cytarabine liposome	chemotherapy	chemotherapy	1-acute myeloid leukemia (t-AML) or AML with myelodysplasia-related changes (AML-MRC) in adults and pediatric patients 1 year and older			2021
US	Wilfin	Eflornithine	Immun	immunotherapy	1-To reduce the risk of relapse in adult and pediatric patients with high-risk neuroblastoma (HRNB) who have demonstrated at least a partial response to prior multiagent, multimodality therapy including anti-GD2 immunotherapy			2023

MA	Medicinal Product (MP)	Active Substance	Category	Category #1	Adults and Paediatric indication	Paediatric indication different from adults or only paediatric	Paediatric indication approval year in EU	Paediatric indication approval year in US
US	Gamifant	Emapalumab-lzsg	monoclonal antibody that binds with affinity to free and receptor-bound IFNγ	immunotherapy	1-Treatment of adult and pediatric (newborn and older) patients with primary hemophagocytic lymphohistiocytosis (HLH)			2018
US	Imbruvica	Ibrutinib	Target therapy by irreversibly binding the protein Bruton's tyrosine kinase (BTK).	target therapy	1-Adult and pediatric patients age 1 year and older with chronic graft versus host disease (cGVHD) after failure of one or more lines of systemic therapy (1.4).			2022
US	Besponsa	inotuzumab ozogamicin.	monoclonal antibody recombinant IgG4 kappa anti-CD22 umanizzato ricombinante	immunotherapy	1-lapsed or refractory CD22-positive B-cell precursor acute lymphoblastic leukemia (ALL) in adult and pediatric patients 1 year and older			2024
US	Azedra	Iobenguane I-131	radiotherapic	other	1-adult and pediatric patients (12 years and older) with iobenguane scan-positive, unresectable, locally advanced or metastatic pheochromocytoma or paraganglioma (PPGL)			2024
US	DANYELZA	Iobenguane I-131	GD2-binding monoclonal antibody i	immunotherapy	1-pediatric patients 1 year of age and older and adult patients with relapsed or refractory high-risk neuroblastoma in the bone or bone marrow			2020
US	GAVRETO	Pralsetinib	selective RET kinase inhibitor	target therapy	1.Treatment of adult and pediatric patients 12 years of age and older with advanced or metastatic RET-mutant or RET fusion-positive medullary thyroid cancer			2020
US	AUGTIRO (EU&FDA)	Repotrectinib	Target therapy. an oral inhibitor of ROS1	target therapy	1-adult and pediatric patients 12 years of age and older with solid tumors that have a neurotrophic tyrosine receptor kinase (NTRK) gene fusion			2024
US	Revuforj	Revumenib	Target therapy binding to menin	target therapy	1-Treatment of relapsed or refractory acute leukemia with a lysine methyltransferase 2A gene (KMT2A) translocation in adult and pediatric patients 1 year and older			2024
US	Elzonris	Tagraxofusp-erzs	a first-in-class CD123-directed immunotherapy	immunotherapy	1-Treatment of blastic plasmacytoid dendritic cell neoplasm (BPDCN) in adults and in pediatric patients 2 years and older			2018

MA	Medicinal Product (MP)	Active Substance	Category	Category #1	Adults and Paediatric indication	Paediatric indication different from adults or only paediatric	Paediatric indication approval year in EU	Paediatric indication approval year in US
US	Tazverik	Tazemetostat	a first-in-class target epigenetic EZH2 inhibitors	other	1-Treatment of adults and pediatric patients aged 16 years and older with metastatic or locally advanced epithelioid sarcoma not eligible for complete resection			2020
US	Ojemda	Tovorafenib	Target therapy. first-in-class targeted epigenetic regulator kinase inhibitor	target therapy		1-Treatment of patients 6 months of age and older with relapsed or refractory pediatric low-grade glioma (LGG) harboring a BRAF fusion or rearrangement, or BRAF V600 mutation		2024
US	Voranigo	Vorasidenib	Target therapy . It is a first-in-class, brain-penetrant, dual inhibitor of the mutant IDH1 and mutant IDH2 enzymes.	target therapy	1-Treatment of adult and pediatric patients 12 years and older with Grade 2 astrocytoma or oligodendroglioma with a susceptible isocitrate dehydrogenase-1 (IDH1) or isocitrate dehydrogenase-2 (IDH2) mutation			2024
US	RYLAZE	asparaginase erwinia chrisantemi ricomb	Enzyme	chemotherapy	1-acute lymphoblastic leukemia (ALL) and lymphoblastic lymphoma (LBL) in adult and pediatric patients			2021
