# Supplementary material for: Scientific and regulatory progress in advancing paediatric oncology drug development in the EU and in the US
Source: Front Med (Lausanne). 2025 Sep 26;12:1642279. doi: 10.3389/fmed.2025.1642279 (PMC12511141; doi:10.3389/fmed.2025.1642279)
Supplement: Supplementary file 2 [file Table_1.DOCX]

**Table 1.** Classification of approved paediatric oncology indications by the EMA and FDA according to tumour type.

|  | **Paediatric tumour type** | **EU-approved**  **N (%)** | **US-approved**  **N (%)** | **Notes^1^** |
| --- | --- | --- | --- | --- |
| **Haematology**  Total EU: 46.9%  Total US: 46.8% | leukaemias | 17 (34.7) | 27 (34.2) |  |
|  | lymphomas | 6 (12.2) | 10 (12.7) |  |
| **Solid tumours**  Total EU: 42.9%  Total US: 44.3% | solid tumors multiple indication | 2 (4.1) | 10 (12.7) | *Repotrectinib* approved in the EU in January 2025 |
|  | neuroblastomas | 1 (2.0) | 3 (3.8) | *Eflornithine* recently submitted for marketing authorisation in the EU |
|  | brain tumor | 6 (12.2) | 7 (8.9) |  |
|  | osteosarcomas and Ewing's sarcomas | 3 (6.1) | 2 (2.5) | *Mifarmurtide* rejected in the US |
|  | melanoma | 3 (6,1) | 3 (3,8) |  |
|  | tyroid cancer  colonrectal carcinoma, other^2^ | 6 (12.2) | 10 (12,7) | *Avelumab* approved in the EU in January 2025 |
| **Supportive**  Total EU: 10.2%  Total US: 5.1% |  | 5 (10.2) | 4 (5.1) |  |
|  | **Total** | **49** | **79** | EU/US total 82 |

^1^ The “Notes” refers to additional information argumented in the discussion

^2^ e.g. Merkel carcinoma

**Table 2.** Classification according to indication category.

| Period | EMA  N (%) | | | FDA  N (%) | | |
| --- | --- | --- | --- | --- | --- | --- |
|  | Adult- driven  indication | Non-adult- driven  indication | TOTAL | Adult- driven  indication | Non-adult- driven  indication | TOTAL |
| 2007-2017 | 21 (95.5) | 1 (4.5) | 22 | 17 (85.0) | 3 (15.0) | 20 |
| 2018-2024 | 19 (70.4) | 8 (29.6)^1^ | 27 | 48 (81.4) | 11 (18.6)^1^ | 59 |
| **2007-2024** | **40 (81.6)** | **9 (18.4)** | **49** | **65 (82.3)** | **14 (17.7)** | **79** |
| Between periods  p-value | 0.024^2^ | | - | 0.712^3^ | | - |
| EMA vs. FDA  p-value | 0.767^4^ | | | | | |

^1^ Of which 5 are ‘only paediatric’

^2^ The chi-square statistic is 5.087.

^3^ The chi-square statistic is 0.136.

^4^ The chi-square statistic is 0.088.

**Table 3.** Classification of MPs approved by EMA and FDA according to drug category

| Period | Drug category: EMA  N (%) | | | | **Tot** | Drug category: FDA  N (%) | | | | **Tot** |
| --- | --- | --- | --- | --- | --- | --- | --- | --- | --- | --- |
|  | Targeted | Immuno | Chemot | other |  | Targeted | Immuno | Chemot | other |  |
| 2007-2017 | 4 (28.6) | 4 (28.6) | 5 (35.7) | 1 | **14** | 5 (33.3) | 5 (33.3) | 4 (26.7) | 1 | **15** |
| 2018-2024 | 12 (57.1) | 5 (23.8) | 3 (14.3) | 1 | **21** | 19 (50.0) | 9 (23.7) | 6 (15.8) | 4 | **38** |
| **2007-2024** | 16 (45.7) | 9 (25.7) | 8 (22.9) | 2 | **35** | 24 (45.3) | 14 (26.4) | 10 (18.9) | 5 | **53** |
| **Time-trend analysis**  targeted drugs *p-value* = 0.254  immunotherapy drugs *p-value* = 0.363  cytotoxic chemotherapy drugs *p-value* = 0.455 | | | | | | | | | | |

**Table 4.** Classification of PIPs according to tumour type.

|  | Paediatric tumour type | PIPs  N |
| --- | --- | --- |
| **Haematology**  Total 98  (45%) | leukaemias | 57 |
|  | lymphomas | 41 |
| **Solid tumor**  Total 111  (51%) | solid tumor multiple indications | 53 |
|  | brain tumors | 17 |
|  | osteosarcomas and Ewing's sarcomas | 5 |
|  | neuroblastomas | 7 |
|  | melanoma | 13 |
|  | other (rabdomiosarcoma and tyroid, colon and breast cancers, etc) | 16 |
| Supportive  (3%) | conditioning, toxicity, post-trasplant ( etc)) | 7 |
|  | **Total** | **216** |

**Table 5.** Classification of PIPs according to indication categories.

| Period | EMA-PIP decision  N (%) | | | | |
| --- | --- | --- | --- | --- | --- |
|  | Adult-driven-  indication | Non-adult-  driven  indication | Only  children  indication | **Total** | *p-value ^1^* |
| 2007-2017 | 27 (65.9) | 14 (34.1) | 0 (0.0) | 41 | 0.003^2^ |
| 2018-2024 | 56 (40.0) | 79 (56.4) | 5 (3.6) | 140 |  |
| Total | 83 (45.9) | 93 (51.4) | 5 (2.8) | 181 | - |

**Table 6.** Classification of PIPs according to drug category. (Immuno, immunotherapy; chemo, chemotherapy)

| Period | Drug category: PIPs | | | | | |
| --- | --- | --- | --- | --- | --- | --- |
|  | Targeted | Immuno | Chemo | other | total | *p-value* |
| 2007-2017 | 6 (18.8) | 5 (15.6) | 14 (43.8) | 7 (21.9) | 32 | < 0.001^1^ |
| 2018-2024 | 55 (39.3) | 41 (29.3) | 21 (15.0) | 23 (16.4) | 140 |  |
| 2007-2024 | 61 (35.5) | 46 (26.7) | 35 (20.3) | 30^2^ (17.4) | 172 | - |

1. The chi-square statistic is 16.078.
