# Supplementary material for: Scientific and regulatory progress in advancing paediatric oncology drug development in the EU and in the US
Source: Front Med (Lausanne). 2025 Sep 26;12:1642279. doi: 10.3389/fmed.2025.1642279 (PMC12511141; doi:10.3389/fmed.2025.1642279)

**Figure 1.** Paediatric oncology MPs, estimated regression line (red line) and 95% confidence interval (blue line): (**a**) Trend in **EU** in 2007 – 2024; (**b**) Trend in **US** in 2007 – 2024.


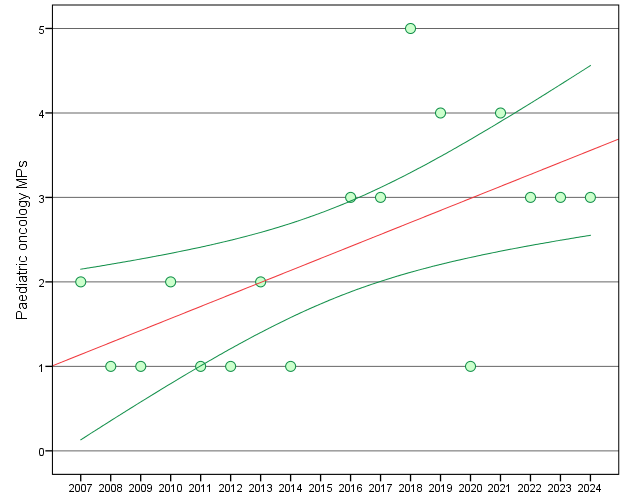

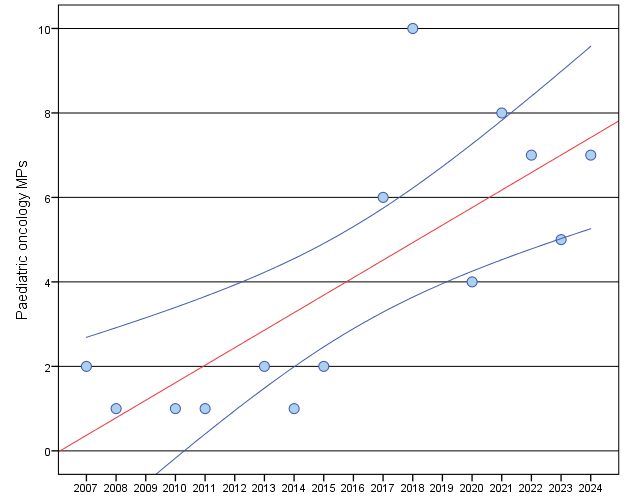


1. b)

**Figure 2**. Trend of PIPs approved between 2008 and 2023 according to tumour type classification: solid tumours (green line) and hematology (blue line).
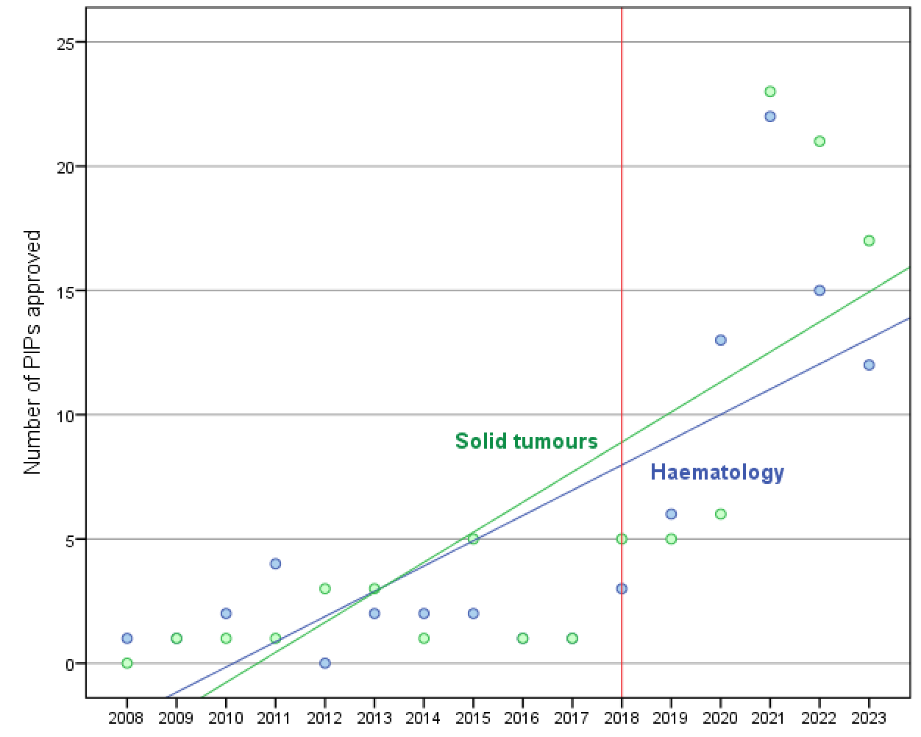

Supplement: Supplementary file 3 [file Table_2.DOCX]
